# Our New York Letter

**Published:** 1890-08

**Authors:** Wm. L. Russell

**Affiliations:** New York


### OUR NEW YORK LETTER.

NEW YORK, July 15, 1890.

An interesting question was raised at the American Pædiatric Society by Dr. W. P. Northrup. He read a paper entitled, "On what early Symptoms may we base the Diagnosis of Tubercular Meningitis during the first two Years of Life?" In elaborating this question he cited several cases, among them one of deep-seated pneumonia with cerebral symptoms, and another of entero-colitis, all of which simulated the disease in question, and concluded by offering us his contribution to the answer. The four symptoms: persistent vomiting, irregular pulse, irregular respiration and apathy.

Dr. C. M. Earle, of Chicago objected to the three symptoms last named, on the ground that they came late, not early. He drew attention to the peculiar projectile character of the vomiting, which occurred without nausea as a most valuable symptom, regarded by him as almost pathognomonic. He considered the following four symptoms of more service than those suggested by Dr. Northrup, *viz.:* A protracted initial stage, when the child was peevish, discontented and restless, with fever, and it was apparent that something unusual was threatened; a history of hereditary taint or traumatism, cerebral vomiting and local spasm.

Dr. Rotch, of Boston, stated emphatically that the diagnosis could not be made in children under two years old. He regarded Dr. Northrup's four symptoms as unsound. Irregular respiration might be reflex; vomiting, even of an explosive character, without nausea might also be reflex, or due to cerebral thrombosis, or without any cerebral disease whatever. Apathy, too, was untrustworthy, for it was impossible to say whether it was due to cerebral diseases or to some other cause.

Dr. Jacobi believes the four symptoms to be valuable only in older children. He had seen irregular pulse in anæmia, and had seen cases of tubercular meningitis go to autopsy without having had at any time the typical vomiting. A slow initial stage was not to be relied on, for he had seen cases develop suddenly with high temperature. Rheumatic or simple meningitis, and the cerebral symptom of nephritis were liable to be mistaken. Dr. Winters regarded the symptoms suggested by Dr. Northrup as positively diagnostic, if properly estimated. It was necessary to eliminate reflex causes, and obtain the family history, and the previous history of the child regarding chances for anto-infection. Then the symptoms must be carefully watched, often hour after hour and during sleep. The irregular pulse was most important of all.

Dr. A. Caillé believed that there were no positive symptoms on which the diagnoses could be based.

Dr. J. H. Fruitnight said that cases were seldom seen in the very early stage; when the physician was called the symptoms suggested by Dr. Northrup were sufficient for a diagnosis.

At the last meeting of the Academic Sections on Obstetrics and Gynæcology, the question of immediate repair of injuries to the pelvic floor during labor was discussed. Dr. I. H. Hance read a paper. He said that the first indication of the injury was the appearance of blood coming from the vagina during the last few minutes preceding the birth of the head. Later the rupture could be felt with the finger. Sometimes the head ploughed through the perineum, making a skin wound in addition to extensive laceration of the posterior vaginal wall. In other cases there were no skin wounds, but the posterior vaginal wall was the seat of a V-shaped contused and lacerated wound. He believes that in every case immediate repair should be undertaken. His method of procedure was to bring the hips to the edge of the bed, the legs being held in a thoroughly flexed position by assistants or by a sling. A vaginal douche having been given, the extent of the injury was accurately defined by advancing the posterior vaginal wall into view by means of two fingers passed into the rectum. The sutures were then introduced from behind forward; that is, beginning at the deep extremity of the wound. He used Nos. 1 and 2 cat-gut, and passed each suture around the laceration. If the wounds were V-shaped, two lines of sutures were used. The edges were carefully coaptated, the skin, if torn, being sewed up last. Iodoform was now dusted over the wound, an iodoform suppository placed in the vagina, and an antiseptic pad applied. The operation caused very little pain, one or two deep sutures being often all required. The cat-gut was absorbed in about seven days. In a hundred cases, which he had sewed up in this way, there were seven failures. He thought that this result was much more satisfactory than to trust to the wound healing by granulation.

Dr. A. P. Dudley approved of the immediate operation. It was his custom to deliver patients in the Sims' position, and thus to watch the perineum. If it ruptured he sewed it up with No. 6 cat-gut, the patient remaining in the position in which she was delivered.

Dr. H. C. Hanks, of the Woman's Hospital, believed that the question of immediate operation could no longer be considered *sub judice*. Antiseptic precautions prevented any additional poison being conveyed to the woman, and so he believed that the operation should always be done. Any suture material was suitable if well prepared, and three sutures were often sufficient.

Dr. H. C. Coe remarked that he used as many sutures as in the secondary operation. On this point there was considerable difference of opinion; some urging that the sutures should not be more than a quarter of an inch apart, others that two were very frequently all that were required.

A recent paper by Dr. R. W. Taylor on unusual modes of infection with syphilis is reviewed editorially in the last number of *The New York Medical Journal*. A number of striking instances are cited. In one the disease was conveyed from a maid to her mistress by means of a towel, and then from the mistress to a friend through the medium of a bolus of chewing gum, which the latter had picked up, thinking it to be her own. Reference is made to the necessity of enquiring in obscure cases regarding certain unnatural practices to which some men are addicted. It was stated that a class of men, victims of sexual perversion, were accustomed to patrol dark and unfrequented streets, and to haunt public parks and places for urination for the purpose of granting favors to any who would submit to their disgusting practices. One of these men was exhibited before the New York Dermatological Society by Dr. Taylor in December, 1888. He was then suffering from a chancre of the tonsil and general syphilitic manifestations, and had probably infected several before his chancre became so painful as to disable him from continuing his beastly pursuit.

Another mode of infection was by post-mortem examinations of the bodies of those who had died while the disease was still active. Dr Taylor had observed two cases, both in physicians.

He also related histories of cases showing infection from a caustic holder, a handkerchief, a bathing suit, a syringe, a pair of drawers, a whistle, a tongue scraper, a razor, a pillow, etc. He had seen, at least, a dozen cases in which the victims, having feared contagion, had abstained from intercourse, and contented themselves with fondling the female genitals. The fingers thus soiled with the secretion of syphilitic lesions, transferred the virus to some other part of the body, generally by scratching or picking. Many chancres of the face in men, very probably, originated in this way.

*A propos* of this subject it may be stated that a hospital for the treatment of venereal and genito-urinary diseases has been recently started in New York. At present, small and comparatively unknown, it will yet become, in all probability, one of the most useful and important in this country. It has been named St. Bartholomews. Its object, as stated in the first annual report, is the treatment of skin and venereal diseases, and all diseases of the genito-urinary tract, with investigations into their etiology and prevention. Among the consulting and visiting physicians and surgeons are Dr. Geo. A. Peters, Dr. Chas. McBurney, Dr. Geo. H. Fox, Dr. M. Allen Starr, Dr. P. A. Morrow and others, whose names are a sufficient guarantee of the quality of work which may be expected. It is believed that this hospital will afford a rich field for the study of syphilis and other venereal diseases, and that the different phases of these disorders will be here presented to the students in such a comprehensive manner as has been hitherto impossible in this country. Besides the clinical material available, a valuable collection of beautiful wax models is being added by Dr. F. J. Leviseur. These casts are so perfect in design and coloring, that rare and particularly interesting cases can be perpetuated in a manner almost equal to life.

Another new feature of the medical world here is a Pasteur Institute. This has been opened by Dr. Paul Gibier, a pupil of Pasteur. Dr. Gibier stated at a recent meeting of the County Medical Association that he had already inoculated sixteen persons. They had all done well. He added that in order to test the sensations produced by these inoculations he had operated on himself and two of his laboratory assistants. The results were similar in all these cases. Four days after the injections some local irritation appeared. Sleep was disturbed, and there was some rise in temperature for ten days. The local symptoms disappeared in three days. The salivary glands and the kidneys were abnormally active. Dizziness and disinclination to work lasted fourteen days. On the tenth day there was some pain in the lumbar region in himself, and in the assistants irregular pains in the nape of the neck, brachial plexus and gluteal and crural regions. On the twelfth day the assistant complained of pain in the head, and on the fourteenth of pains at the point of inoculation. Now, at the end of a month and a half all were perfectly well.

WM. L. RUSSELL.

